# Lineage-specific exosomes promote the odontogenic differentiation of human dental pulp stem cells (DPSCs) through TGFβ1/smads signaling pathway via transfer of microRNAs

**DOI:** 10.1186/s13287-019-1278-x

**Published:** 2019-06-13

**Authors:** Xiaoli Hu, Yingqun Zhong, Yuanyuan Kong, Yanan Chen, Junming Feng, Jianmao Zheng

**Affiliations:** 1 Department of Operative Dentistry and Endodontics, Guanghua School of Stomatology, Affiliated Stomatological Hospital, Sun Yat-sen University, Guangzhou, 510055 Guangdong China; 2 Guangdong Provincial Key Laboratory of Stomatology, Sun Yat-sen University, Guangzhou, Guangdong China; 3 Key Laboratory of Oral Medicine, Guangzhou Institute of Oral Disease, Stomatology Hospital of Guangzhou Medical University, Guangzhou, Guangdong China; 4 Department of Endodontics, Stomatology Hospital of Guangzhou Medical University, Guangzhou, Guangdong China

**Keywords:** Exosomes, TGFβ pathway, MicroRNAs, Odontogenesis, DPSCs

## Abstract

**Background:**

Exosomes derived from dental pulp stem cells (DPSCs) can be used as biomimetic tools to induce odontogenic differentiation of stem cells, but the regulatory mechanisms and functions of exosome-encapsulated microRNAs are still unknown. The present study aimed to clarify the role of microRNAs contained in the exosomes derived from human DPSCs and their potential signaling cascade in odontogenic differentiation.

**Methods:**

Exosomes were isolated from human DPSCs cultured undergrowth and odontogenic differentiation conditions, named UN-Exo and OD-Exo, respectively. The microRNA sequencing was performed to explore the microRNA profile contained in UN-Exo and OD-Exo. Pathway analysis was taken to detect enriched pathways associated with the predicted target genes of microRNAs. The regulatory roles of a highly expressed microRNA in OD-Exo were investigated through its inhibition or overexpression (miRNA inhibitors and miRNA mimics). Automated western blot was used to identify the function of exosomal microRNA and the roles of TGFβ1/smads pathway in odontogenic differentiation of DPSCs. A luciferase reporter gene assay was used to verify the direct target gene of exosomal miR-27a-5p.

**Results:**

Endocytosis of OD-Exo triggered odontogenic differentiation of DPSCs by upregulating DSP, DMP-1, ALP, and RUNX2 proteins. MicroRNA sequencing showed that 28 microRNAs significantly changed in OD-Exo, of which 7 increased and 21 decreased. Pathway analysis showed genes targeted by differentially expressed microRNAs were involved in multiple signal transductions, including TGFβ pathway. 16 genes targeted by 15 differentially expressed microRNAs were involved in TGFβ signaling. Consistently, automated western blot found that OD-Exo activated TGFβ1 pathway by upregulating TGFβ1, TGFR1, p-Smad2/3, and Smad4 in DPSCs. Accordingly, once the TGFβ1 signaling pathway was inhibited by SB525334, protein levels of p-Smad2/3, DSP, and DMP-1 were significantly decreased in DPSCs treated with OD-Exo. MiR-27a-5p was expressed 11 times higher in OD-Exo, while miR-27a-5p promoted odontogenic differentiation of DPSCs and significantly upregulated TGFβ1, TGFR1, p-Smad2/3, and Smad4 by downregulating the inhibitory molecule LTBP1.

**Conclusions:**

The microRNA expression profiles of exosomes derived from DPSCs were identified. OD-Exo isolated under odontogenic conditions were better inducers of DPSC differentiation. Exosomal microRNAs promoted odontogenic differentiation via TGFβ1/smads signaling pathway by downregulating LTBP1.

**Electronic supplementary material:**

The online version of this article (10.1186/s13287-019-1278-x) contains supplementary material, which is available to authorized users.

## Introduction

Clinically relevant dental pulp tissue regeneration can serve as a treatment to replace existing root canal therapy used to treat necrotic permanent teeth [1]. Regenerative endodontic treatment is attempted by using a variety of mesenchymal stem cells (MSCs), growth factors, and biomaterials [2, 3]. Exosomes are reported as an ideal biomaterial in regenerative endodontic treatment, with the properties of immunomodulation [4, 5], promoting angiogenesis [6] and inducing differentiation of stem cells [7, 8].

Exosomes are nano-sized vesicles ranging from 30 to 150 nm in diameter, which are secreted by many cell types to mediate intercellular communication [9]. The cargo of exosomes is shown to contain both ubiquitous and cell type-specific biological molecules such as protein, mRNA, and microRNA [9]. It has been revealed that exosomal microRNAs are transferred between cells and can regulate the post-transcriptional gene expression in recipient cells [10]. microRNAs can negatively regulate gene expression at the post-transcriptional level by binding to their target mRNAs through base pairing to the 3′-untranslated region (UTR), causing translational repression of the mRNA [11]. Exosomal microRNAs have been shown to be critical components in stem cell differentiation. For example, exosomal miR-320c enhanced chondrogenic differentiation of bone marrow MSCs by upregulating SOX9 and downregulating MMP13 expression [12]. Similarly, exosomal miR-let-7 has been shown to initiate the osteogenic differentiation of MSCs [13].

Exosomes derived from dental pulp stem cells (DPSCs) can be used as biomimetic tools to induce odontogenic differentiation of stem cells during dental pulp regeneration [14]; however, the function and regulatory mechanism of microRNAs encapsulated in exosomes derived from DPSCs is still unknown. To date, no study has been conducted on the microRNA expression profiles of exosomes derived from human DPSCs. The present study aimed to clarify the role of microRNAs, encapsulated in the exosomes from human DPSCs, and their potential signaling cascade in odontogenic differentiation.

## Materials and methods

### Isolation and culture of human DPSCs

All experimental protocols were approved by the Ethics Committee of Sun Yat-sen University. Human DPSCs were harvested from healthy pulp tissues isolated from caries-free teeth of patients (5 females, age 24~35 years; 5 males, age 22~36 years) undergoing extraction of fully erupted third molars. Healthy pulp tissues were digested for isolation of DPSCs as described previously [15]. Cells were cultured at 37 °C, in a 5% CO_2_ incubator, using а-MEM supplemented with 10% FBS (GIBCO, USA) as a growth medium, 10 mg/ml streptomycin, and 10 U/ml of penicillin (Sigma, USA). Experiments were performed with DPSCs from passages 3 to 7.

### Investigation of DPSC surface markers

100 *μ*l DPSCs at a concentration of 1 × 10^6^ cells/ml were stained by 5 *μ*l of each of the following human antibodies: CD45-PE, CD73-PE, CD90-APC, and CD166-PE (BD, USA). The samples were incubated at 37 °C for 30 min, centrifuged, washed twice with PBS, and examined by flow cytometry (BD, USA).

### Determination of DPSC differentiation capacity

We determined the multi-potential differentiation of DPSCs into osteoblasts, adipocytes, and chondrocytes in vitro. To explore the potential of differentiation into osteoblasts, DPSCs were induced for 14 days in osteogenic medium supplemented with 100 nmol dexamethasone, 10 mmol β-glycerophosphate, and 0.2 mmol l-ascorbic acid (Sigma, USA), osteogenic differentiation was measured by Alizarin Red S staining. To verify the adipogenic differentiation potential, DPSCs were induced for 27 days in adipogenic medium supplemented with 0.5 *μ*M isobutyl-methylxanthine, 50 *μ*M indomethacin, 0.5 *μ*M dexamethasone, and 5 *μ*g/mL insulin (Sigma, USA), adipogenic differentiation was determined by Oil Red O staining. For chondrogenic differentiation, the cell pellets were prepared for a three-dimensional culture system. Approximately 4 × 10^5^ cells were placed in a 15-ml polypropylene tube and centrifuged at 500 g for 5 min. The cells were cultured in human mesenchymal stem cell chondrogenic differentiation medium (Cyagen, USA) for 28 days, in the presence of 10 ng/mL recombinant human TGF-b3. Alcian blue staining was utilized to examine the cartilage nodules.

### Isolation and identification of exosomes

Exosomes were isolated from the culture medium of DPSCs cultured in the presence of either growth (UN-Exo) or odontogenic differentiation media (OD-Exo) for a period of 10 days. Exosomes were isolated as per previously published protocols [16, 17]. Briefly, 2 days prior to isolation, the cell cultures were washed in serum-free PBS and cultured for 48 h in serum-free а-MEM. When odontogenic media were used, the serum-free а-MEM was supplemented with the odontogenic media cocktail of 100 nmol dexamethasone, 10 mmol β-glycerophosphate, and 0.2 mmol l-ascorbic acid (Sigma, USA). The exosomes from the culture medium were isolated using the Exo-spin (Cell Guidance, UK) exosome isolation reagent as per the manufacturer’s protocol. The exosome protein concentration was quantified with a BCA Protein Assay Kit (Bocai, Shanghai, China). The exosomal markers CD9 and CD63 (Affinity Biosciences, USA) in the UN-Exo and OD-Exo were measured by automated western blot analysis.

### Transmission electron microscopy (TEM)

TEM was used to identify the presence of UN-Exo and OD-Exo. 10 *μ*l exosomes suspension was placed on to formvar/carbon-coated nickel TEM grids and incubated for 30 min. The grids were then washed, dried, and imaged using an H-7650 transmission electron microscope (HITACHI, Japan) to identify the morphology of exosomes.

### Fluorescent labeling of exosomes

UN-Exo and OD-Exo were collected from supernatants of DPSC and isolated by ultracentrifugation and sucrose cushion centrifugation. Briefly, cell culture supernatant (48 h, serum-free medium) was cleared (2 × 10 min, 500×*g*; 1 × 20 min, 2000×*g*; 1 × 30 min, 10,000×*g*), centrifuged (90 min, 100,000×*g*), washed (PBS, 90 min, 100,000×*g*), and further purified by sucrose-gradient centrifugation. 1 *μ*l PKH26 (Sigma-Aldrich, St Louis, MO) was added to 250 *μ*l diluent C, which was then immediately mixed with the exosomes by pipetting. After 5 min of incubation at room temperature, the staining was stopped by the addition of an equal volume of exosome-free FBS. The exosomes were harvested and washed twice with PBS by centrifugation (100,000 g for 1 h) and resuspended in 100 *μ*l exosome-free culture medium. The PKH26-labeled exosomes were added to the DPSCs and incubated for 24 h. Then, the cells were washed 3 times with PBS, fixed with 4% paraformaldehyde for 10 min, and stained with 4, 6-diamidino-2-phenylindole for 5 min. Confocal laser scanning microscopy (Zeiss, Oberkochen, Germany) was used to visualize the endocytosis of UN-Exo and OD-Exo by DPSCs.

### Exosome-mediated odontogenic differentiation of DPSCs

DPSCs were seeded into 6-well plates at an initial density of 1 × 10^5^ cells/well and incubated for 48 h with exosomes isolated from DPSCs cultured for 10 days using growth media (UN-Exo, 30 *μ*g/ml) as well as odontogenic differentiation media (OD-Exo, 30 *μ*g/ml), the growth media supplemented with 10% exosome-free FBS was used as control medium. Odontogenic differentiation was measured by automated western blot to explore the protein expression of ALP, RUNX2, odontoblast-specific marker DSP, and DMP-1.

### Automated western blot analysis

The automated western blot was performed using Simple Wes (Protein Simple, USA) following the manufacturer’s protocol. Briefly, 1.5 *μ*g of protein from the cell lysates or exosomes was added to the standard fluorescent mastermix, then was loaded into corresponding wells of the prefilled Wes assay plate, along with antibody diluent (Protein Simple, USA), anti-DSP (Santa Cruz, USA), anti-DMP1, anti-RUNX2, anti-ALP, anti-CD9, anti-CD63, anti-TGFβ1, anti-TGFR1, anti-Smad2/3, anti-p-Smad2/3, anti-Smad4, anti-β-Tublin (Affinity, USA), anti-LTBP1 (Affinity, USA), anti-rabbit secondary antibody (Protein Simple, USA), and Streptavidin-HRP, followed by luminal peroxide mix. The imaging and analysis were done with compass software (Protein Simple, USA).

### microRNA sequencing

MicroRNAs of 18–30 nucleotides (nt) were obtained from 100 *μ*g of total RNA isolated from exosomes using 15% denaturing polyacrylamide gel electrophoresis (PAGE). After PCR amplification, the products were purified and submitted for sequencing via an Illumina Hi-Seq 2000 platform. Library preparation and microRNA sequencing were performed by RiboBio Ltd. (Guangzhou, China). Differentially expressed microRNAs with 2 fold change in expression (*p* < 0.05) were analyzed. Predicted microRNA target genes were detected using four publicly available bioinformatics tools (TargetScan, miRTarBase, miRDB, and miRWalk databases). Gene Ontology (GO, http://www.geneontology.org) analysis and Kyoto Encyclopedia of Genes and Genomes (KEGG, http://www.genome.jp/kegg/pathway.html) Pathway analysis were performed to detect molecular functions, biological processes, and pathways associated with the predicted microRNA target genes.

GO analysis, all the targeted genes of miRNAs were input into the Gene Ontology database, and each term number of genes was calculated. Compared with the whole genome background, the hypergeometric test was used to find out the GO terms that were significantly enriched in the targeted genes of miRNAs. The formula is as follows: $p=1-{\sum}_{i=0}^{m-1}\frac{\left(\genfrac{}{}{0pt}{}{M}{i}\right)\left(\genfrac{}{}{0pt}{}{N-M}{n-i}\right)}{\left(\genfrac{}{}{0pt}{}{N}{n}\right)}$, where *N* is the number of all genes with GO annotation, *n* is the number of targeted genes of miRNAs in *N*, *M* is the number of all genes that are annotated to certain GO terms, *m* is the number of targeted genes of miRNAs in *M*. The calculated *p* value goes through Bonferroni correction, taking corrected *p* value < 0.05 as a threshold. GO terms fulfilling this condition are defined as significantly enriched GO terms in targeted genes of miRNAs. This analysis is able to recognize the main biological functions that targeted genes of miRNA exercise.

KEGG, the major public pathway-related database, is used to perform pathway enrichment analysis of targeted genes of miRNAs. This analysis identifies significantly enriched metabolic pathways or signal transduction pathways in targeted genes of miRNAs comparing with the whole genome background. The calculating formula is the same as that in the GO analysis. Here, *N* is the number of all genes that with KEGG annotation, *n* is the number of targeted genes of miRNAs in *N*, *M* is the number of all genes annotated to specific pathways, and *m* is the number of targeted genes of miRNAs in *M*.

### Real-time PCR

Total RNA was extracted from cells by RNA extraction kit (Qiagen, China). Then, 2 *μ*g of total RNA was reversely transcribed into cDNA using a reverse transcription polymerase chain reaction (RT-PCR) system (Promega, USA). The qRT-PCR was performed by SYBR-Green PCR kit (Qiagen, China) according to the manufacturer’s instructions on a LightCycler 480 (Roche, USA). The data are representative of three independent experiments, and the relative microRNA expression was determined using the comparative Ct (ΔΔCt) method. The sequences of the primers are shown in Additional file 1: Table S1.

### Determination and inhibition of TGFβ1/smads signaling pathway

For evaluation of the involvement of TGFβ1/smads signaling pathway, DPSCs were seeded into 6-well plates at an initial density of 1 × 10^5^ cells/well and were incubated for 48 h with 30 *μ*g/ml UN-Exo and 30 *μ*g/ml OD-Exo, the growth media supplemented with 10% exosomes-free FBS was used as the control medium. TGFβ1/smads signaling pathway was inhibited by 10 *μ*M inhibitor SB525334 (Selleck, USA) for 48 h.

### Transfection of miR-27a-5p mimics and inhibitor

DPSCs were treated with OD-Exo in 6-well culture plates and transfected with the miR-27a-5p mimics and inhibitor using Lipofectamine 2000 (Invitrogen, USA) according to the manufacturer’s instructions. DPSCs were harvested after 48 h.

### Dual-luciferase reporter assay

A luciferase reporter gene assay was used to verify whether LTBP1 was the direct target gene of miR-27a-5p. Luciferase reporter constructs encoding the wild-type 3′-UTRs of LTBP1 (LTBP1-WT) or mutant 3′-UTRs of AXL (LTBP1-MUT) were synthesized. The 3′-UTR luciferase vector (150 ng) was co-transfected into cells with either miR-27a-5p mimic or miR-27a-5p mimic-control using Lipofectamine 2000 (Invitrogen). After incubation for 48 h, the cells were collected and lysed, and their luciferase activities were detected by the Dual-Luciferase Reporter Assay Kit (Beyotime Biotechnology, Shanghai, China) according to the manufacturer’s protocol.

### Statistical analysis

Each experiment was repeated three times. All values were expressed as the mean ± SD and were evaluated by the independent samples *t* test using SPSS 17.0 (SPSS Inc., USA). *p* < 0.05 was considered statistically significant.

## Results

### Characterization of DPSCs

**Fig. 1:**
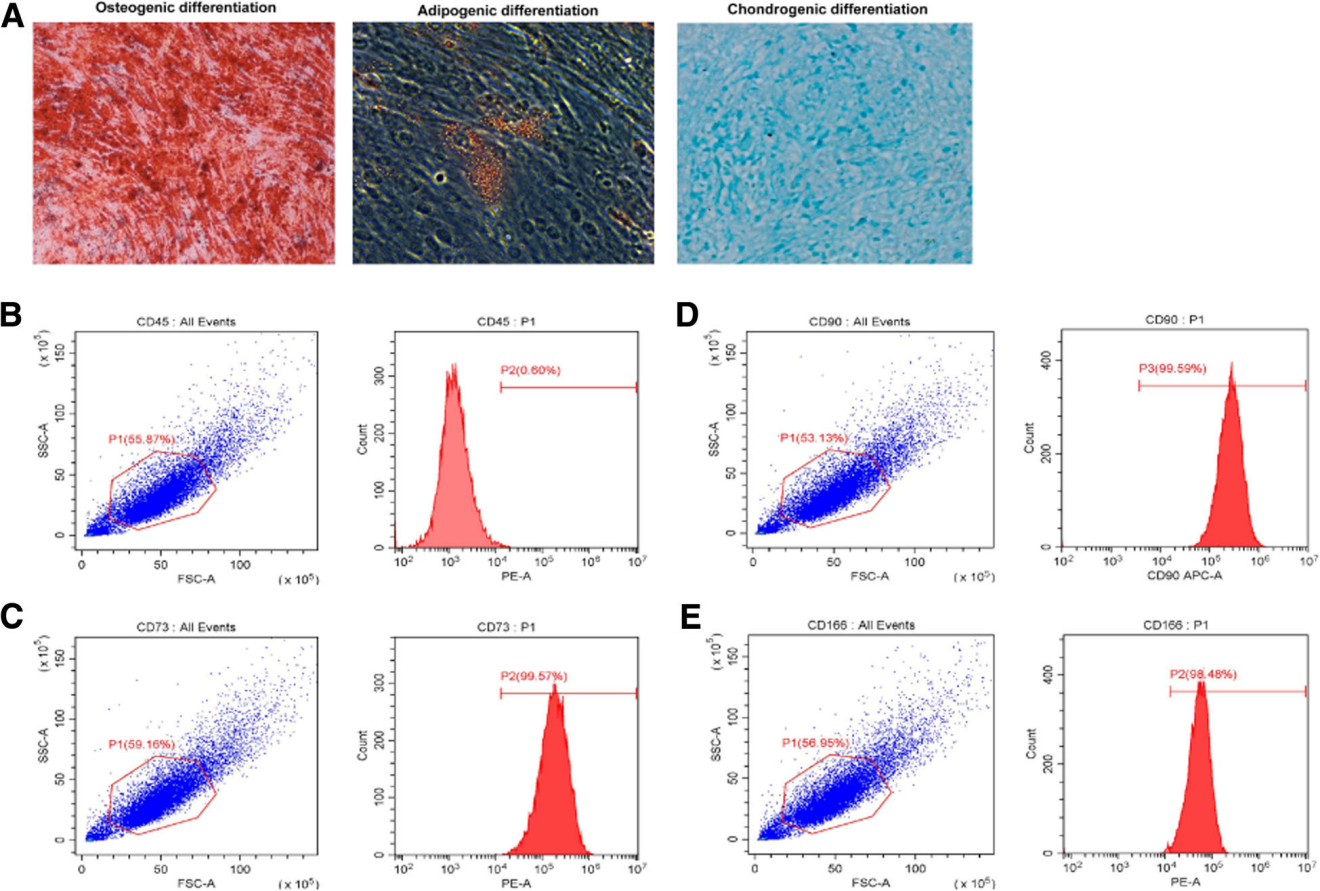
Characterization of DPSCs. **a** DPSCs had the potential of differentiation into osteoblasts, adipocytes, and chondrocytes. **b**–**e** DPSCs expressed high levels of the mesenchymal stem cell marker CD73, CD90, and CD166, but expressed low levels of the hematopoietic cell marker CD45

The results showed that DPSCs had the potential of differentiation into osteoblasts, adipocytes, and chondrocytes (Fig. 1a), indicating the multi-lineage differentiation potential of DPSCs. DPSCs expressed high levels of the mesenchymal stem cell marker CD73 (Fig. 1b), CD90 (Fig. 1c), and CD166 (Fig. 1d), but expressed low levels of the hematopoietic cell marker CD45(Fig. 1e).

### Endocytosis of UN-Exo and OD-Exo by DPSCs

**Fig. 2:**
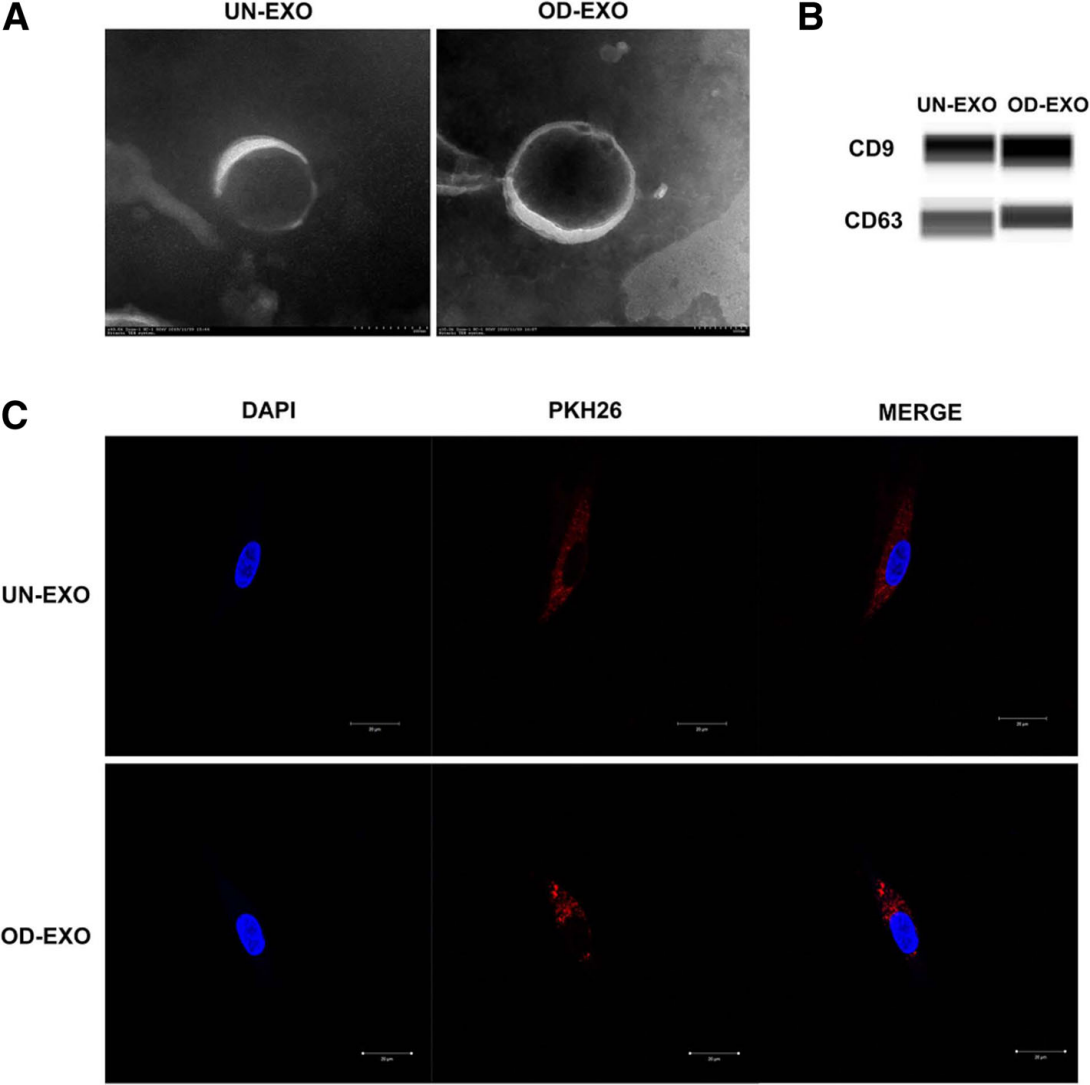
Endocytosis of UN-Exo and OD-Exo by DPSCs. **a** The morphology of UN-Exo and OD-Exo was determined by transmission electron microscopy. **b** Automated western blot analysis revealed that exosomal markers CD9 and CD63 were expressed in the UN-Exo and OD-Exo. **c** Endocytosis of exosomes by DPSCs was visualized by fluorescent labeling with PKH26

To characterize the presence of exosomes in the isolates, the bilayer membrane and “saucerlike” appearance of representative exosomes were examined by TEM, which verified the presence of UN-Exo and OD-Exo ranging from 30 to 150 nm in diameter (Fig. 2a). Automated western blot analysis revealed that exosomal markers CD9 and CD63 were expressed in the UN-Exo and OD-Exo (Fig. 2b). To confirm whether UN-Exo and OD-Exo could be taken up by DPSCs, the isolated UN-Exo and OD-Exo were labeled with PKH26, and DPSC cultures were incubated with the labeled exosomes at 37 °C. After 24 h, PKH26-labeled UN-Exo and OD-Exo were taken up by DPSCs into the cytoplasm (Fig. 2c).

### Endocytosis of OD-Exo by DPSCs triggered odontogenic differentiation

**Fig. 3:**
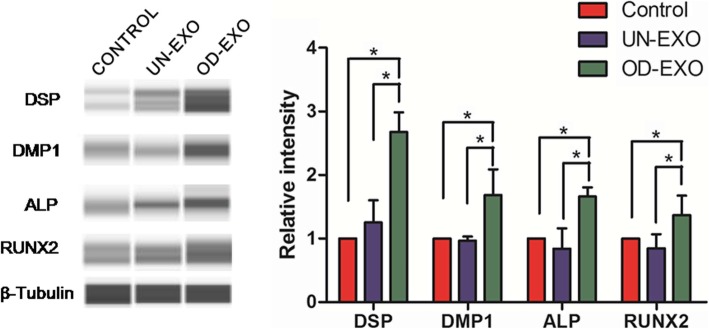
Endocytosis of OD-Exo by DPSCs triggered odontogenic differentiation. Endocytosis of OD-Exo isolated under odontogenic conditions triggered odontogenic differentiation of DPSCs by upregulating protein expressions of DSP, DMP-1, ALP, and RUNX2, when compared to control group (without exosomes) and UN-Exo group (*p* < 0.05). And DPSCs treated with UN-Exo only expressed higher protein level of DSP than the control group (*p* < 0.05), but no significant differences in DMP-1, ALP, and RUNX2

We evaluated whether the endocytosis of exosomes triggered odontogenic differentiation of DPSCs by affecting the expression of regulatory proteins. When DPSCs were treated with OD-Exo for 48 h, the protein expressions of DSP, DMP-1, ALP, and RUNX2 significantly increased, compared to the control group and UN-Exo treated group (*p* < 0.05). However, DPSCs treated with UN-Exo only expressed a higher level of DSP than the control group (*p* < 0.05), but no significant differences in DMP-1, ALP, and RUNX2 (Fig. 3). These results showed that OD-Exo isolated under odontogenic conditions are better inducers of DPSCs differentiation.

### MicroRNA profiles of UN-Exo and OD-Exo

**Fig. 4:**
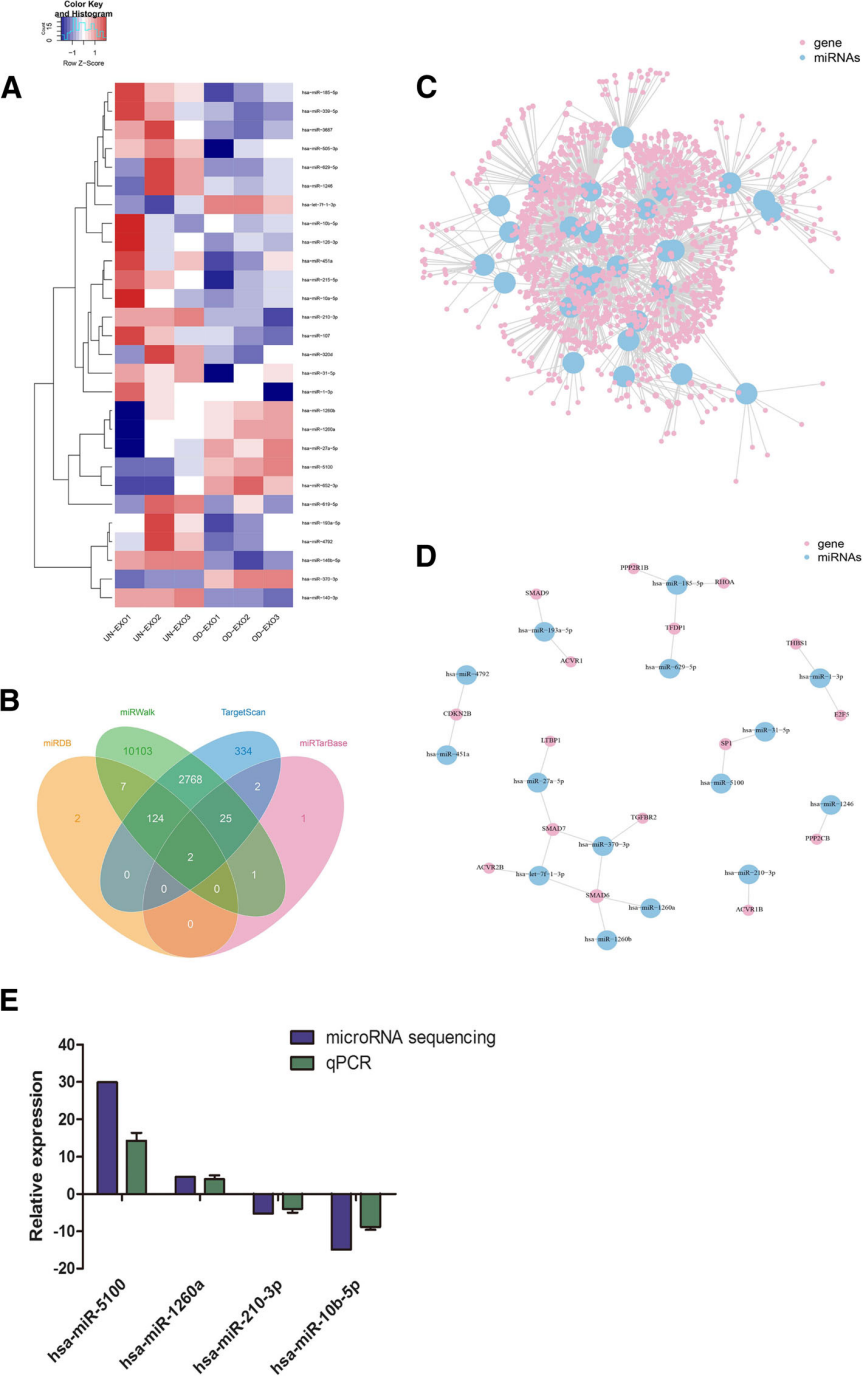
microRNA profiles of UN-Exo and OD-Exo via microRNA sequencing. **a** MicroRNA levels in OD-Exo significantly changed when compared with that in UN-Exo, 28 microRNAs significantly changed, of which 7 increased and 21 decreased. **b** Four bioinformatics tools (TargetScan, miRTarBase, miRDB, and miRWalk) were used to analyze genes targeted by differentially expressed microRNAs, for example, hsa-miR-27a-5p. **c** All genes targeted by differentially expressed microRNAs were shown in the mRNA-microRNA network. **d** mRNA-microRNA network showed 16 genes in TGFβ signaling were targeted by 15 differentially expressed microRNAs. **e** Consistent with the microRNA sequencing, hsa-miR-5100 and hsa-miR-1260a were increased; hsa-miR-210-3p and hsa-miR-10b-5p were decreased by qPCR analysis

As exosomes transfer microRNAs between cells, post-transcriptional gene expression in recipient cells can be regulated by microRNAs contained in exosomes [18]. Thus, we hypothesized that this process may be exploited by DPSCs to promote odontogenic differentiation. To explore this possibility, we analyzed the microRNA profiles of UN-Exo and OD-Exo via Ion Torrent/MiSeq sequencing. The results showed microRNA levels in OD-Exo significantly changed when compared with that in UN-Exo. There were 28 microRNAs significantly changed in OD-Exo isolated under odontogenic conditions, of which 7 microRNAs increased (miR-5100, miR-27a-5p, miR-652-3p, miR-1260a, miR-1260b, let-7f-1-3p, and miR-370-3p) and 21 microRNAs decreased (miR-193a-5p, miR-4792, miR-505-3p, miR-629-5p, miR-140-3p, miR-185-5p, miR-146b-5p, miR-339-5p, miR-1246, miR-107, miR-320d, miR-451a, miR-215-5p, miR-126-3p, miR-3687, miR-31-5p, miR-210-3p, miR-1-3p, miR-10a-5p, miR-10b-5p, and miR-619-5p) (Fig. 4a, Table 1). The qRT-PCR analysis showed that miR-5100 and miR-1260a levels in OD-Exo increased, while miR-210-3p and miR-10b-5p decreased, which were consistent with the microRNA sequencing (Fig. 4e).

**Table 1:** There were 28 microRNAs significantly changed in OD-Exo isolated under odontogenic conditions, of which 7 increased and 21 decreased

miRNA_ID	Up/down	Fold change	Significance
hsa-miR-5100	Up	29.91	**
hsa-miR-27a-5p	Up	11.42	**
hsa-miR-652-3p	Up	10.62	*
hsa-miR-1260a	Up	4.65	**
hsa-miR-1260b	Up	4.00	**
hsa-let-7f-1-3p	Up	2.61	**
hsa-miR-370-3p	Up	2.54	**
hsa-miR-193a-5p	Down	2.09	*
hsa-miR-4792	Down	2.13	*
hsa-miR-505-3p	Down	2.27	*
hsa-miR-629-5p	Down	2.35	*
hsa-miR-140-3p	Down	2.72	**
hsa-miR-185-5p	Down	2.79	*
hsa-miR-146b-5p	Down	2.94	**
hsa-miR-339-5p	Down	3.08	*
hsa-miR-1246	Down	3.56	*
hsa-miR-107	Down	3.65	*
hsa-miR-320d	Down	3.79	*
hsa-miR-451a	Down	4.20	*
hsa-miR-215-5p	Down	4.39	**
hsa-miR-126-3p	Down	4.44	*
hsa-miR-3687	Down	4.64	**
hsa-miR-31-5p	Down	4.93	*
hsa-miR-210-3p	Down	5.23	**
hsa-miR-1-3p	Down	9.15	*
hsa-miR-10a-5p	Down	11.39	**
hsa-miR-10b-5p	Down	14.91	**
hsa-miR-619-5p	Down	17.70	**

*p < 0.05, **p < 0.01

### Pathway and GO analysis of genes targeted by differentially expressed microRNAs

**Fig. 5:**
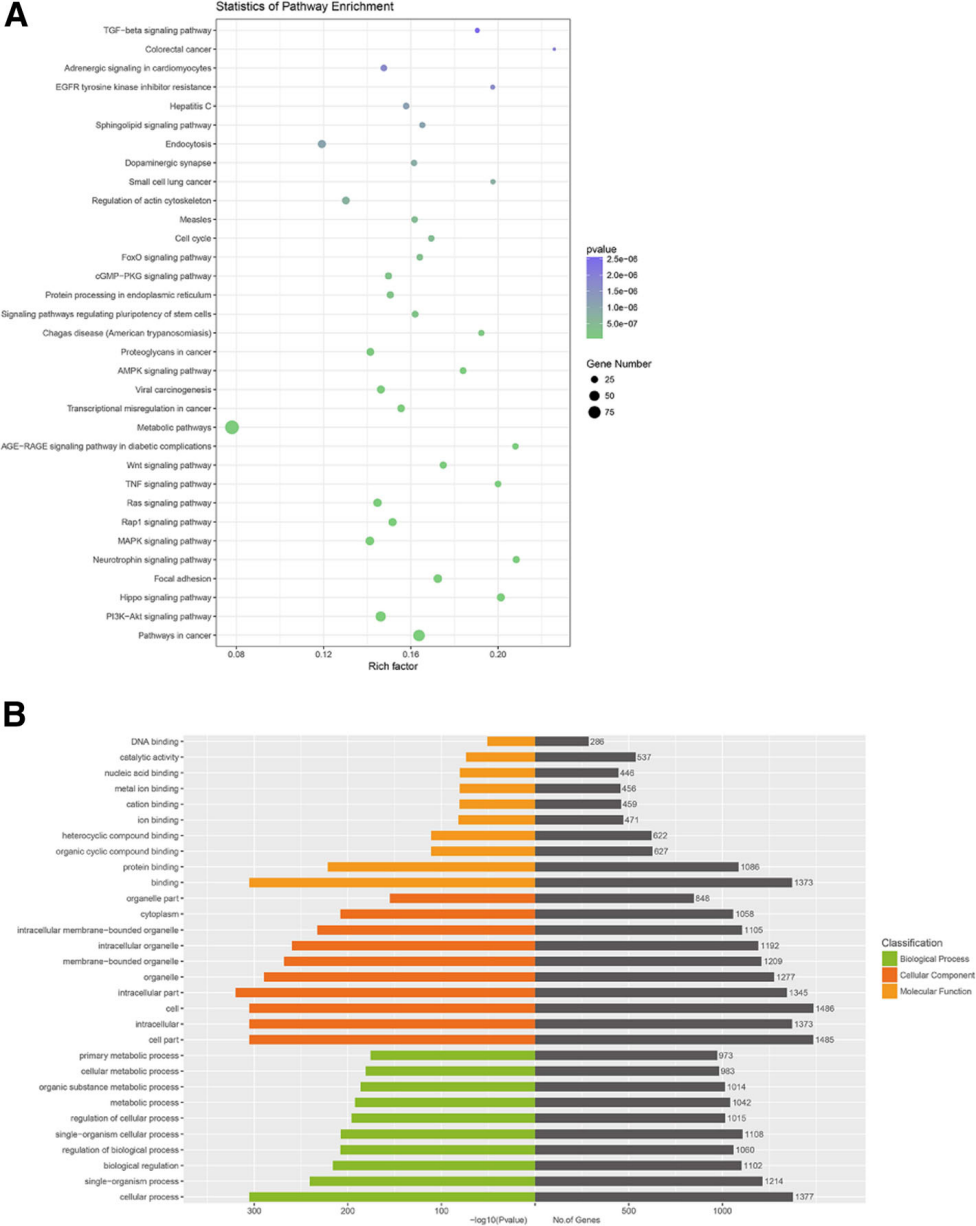
Pathway and GO analysis of genes targeted by differentially expressed microRNAs. **a** Pathway analysis showed that targeted genes involved in multiple signal transductions, including TGFβ signaling pathway. **b** GO analysis of targeted genes showed that the most significant biological processes consisted of DNA binding, catalytic activity, cellular metabolic process, and regulation of the cellular process

Four publicly available bioinformatics tools (TargetScan, miRTarBase, miRDB, and miRWalk) were used to analyze genes targeted by differentially expressed microRNAs, for example, miR-27a-5p (Fig. 4b). All genes targeted by differentially expressed microRNAs were shown in Fig. 4c and Additional file 2: Table S2. Pathway analysis showed targeted genes involved in multiple signal transductions, including TGFβ signaling pathway (Fig. 5a). 16 genes in TGFβ signaling targeted by 15 differentially expressed microRNAs were shown in Fig. 4d. GO analysis of targeted genes showed that the most significant biological processes consisted of DNA binding, catalytic activity, cellular metabolic processes, and regulation of cellular processes (Fig. 5b).

### Exosomal microRNAs promoted odontogenic differentiation via TGFβ1/smads signaling pathway by downregulating LTBP1

**Fig. 6:**
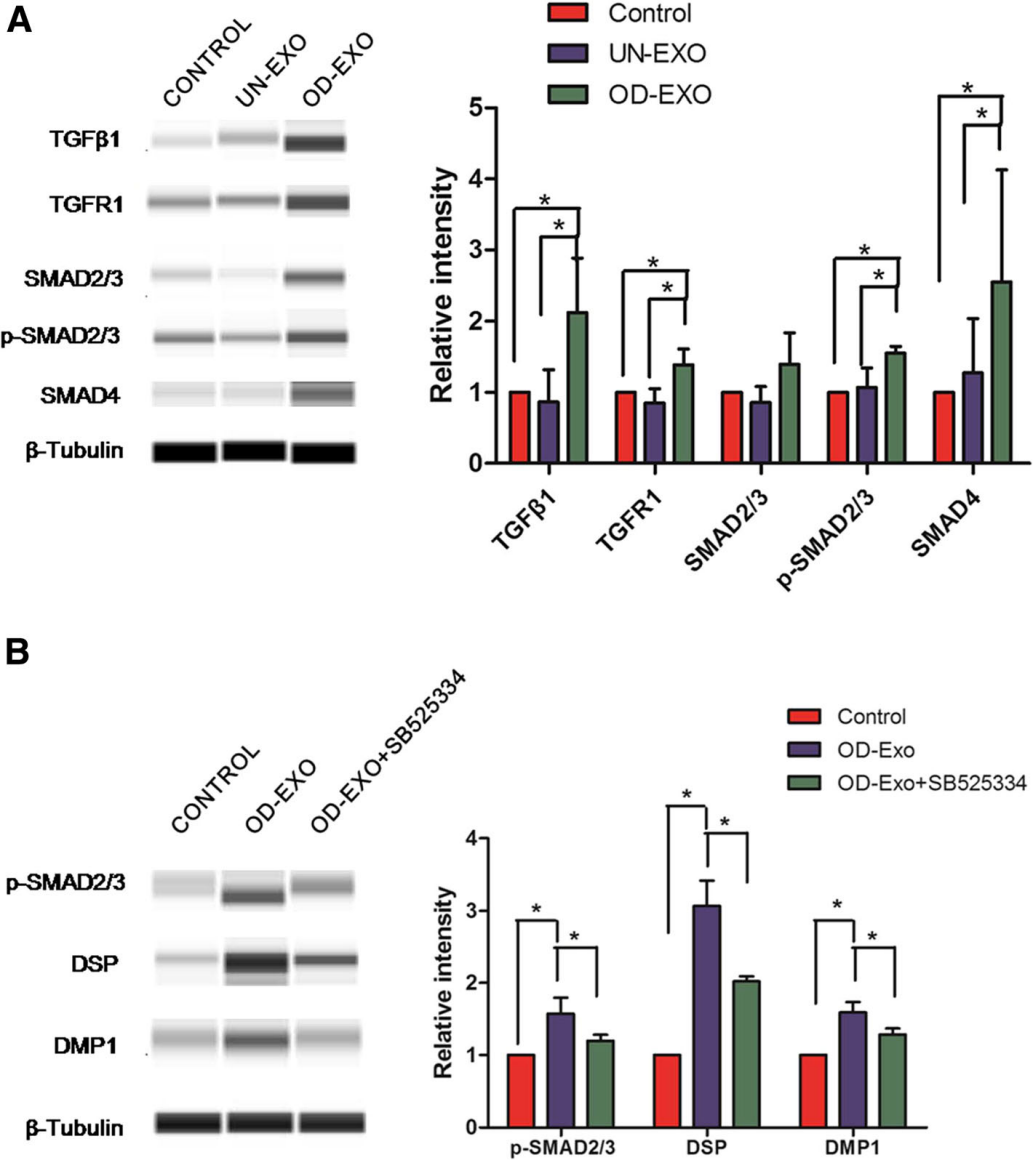
OD-Exo promoted odontogenic differentiation via TGFβ1/smads signaling pathway. **a** Consistent with pathway analysis, automated western blot analysis found significantly upregulated protein expressions of TGFβ1, TGFR1, p-Smad2/3, and Smad4 in DPSCs treated with OD-Exo, when compared to the control group and UN-Exo-treated group. **b** In accordance, once we inhibited the TGFβ1 signaling pathways by SB525334, protein levels of p-Smad2/3, DSP, and DMP-1 were significantly reduced in DPSCs treated with OD-Exo

To confirm the results of pathway analysis as to whether TGFβ signaling was activated by exosomal microRNAs encapsulated in OD-Exo, we performed the automated western blot to evaluate the key proteins in TGFβ signaling. Consistent with pathway analysis, automated western blot found that OD-Exo activated TGFβ1 pathway by upregulating TGFβ1, TGFR1, p-Smad2/3, and Smad4 in DPSCs, compared to control group and UN-Exo-treated group (Fig. 6a). Accordingly, once we inhibited the TGFβ1 signaling pathways by SB525334, protein levels of p-Smad2/3, DSP, and DMP-1 were significantly reduced in DPSCs during odontogenic differentiation induced by OD-Exo for 48 h (Fig. 6b).

**Fig. 7:**
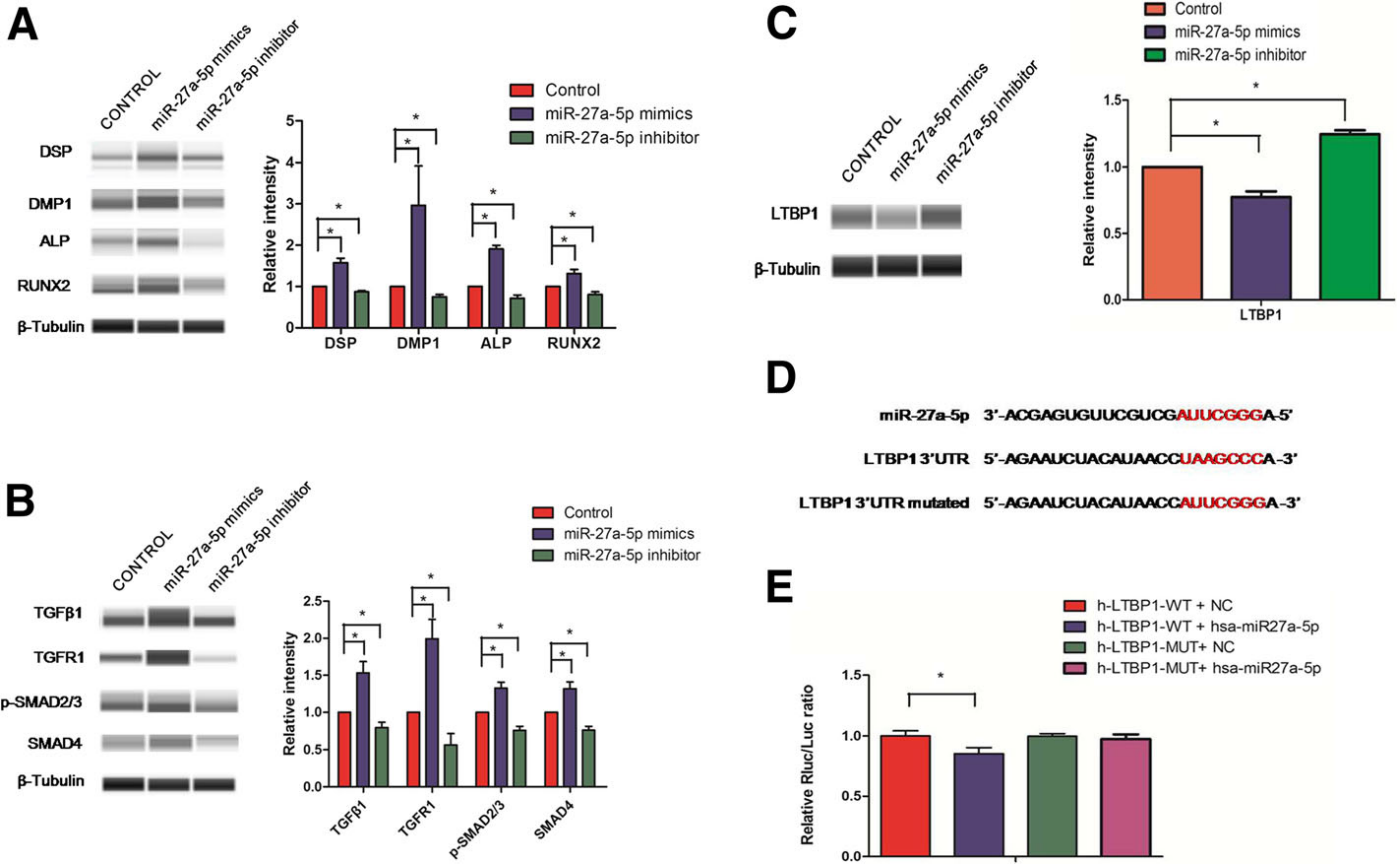
Exosomal miR-27a-5p regulated odontogenic differentiation via TGFβ1/smads signaling pathway by downregulating LTBP1. **a** miR-27a-5p mimics promoted odontogenic differentiation of DPSCs by upregulating the protein expressions of DSP, DMP-1, ALP, and RUNX2. **b** miR-27a-5p mimics activated TGFβ1/smads signaling pathway by increasing TGFβ1, TGFR1, p-Smad2/3, and Smad4 proteins. **c** The western blot showed LTBP1 was downregulated bymiR-27a-5p mimics. **d** The predicted miRNA binding sites in the 3′-UTR of LTBP1. **e** Luciferase reporter assay found that miR-27a-5p could significantly reduce LTBP1-WT luciferase activity and that miR-27a-5p had no effect on the luciferase activity of LTBP1-MUT group

MiR-27a-5p was expressed 11 times higher in OD-Exo isolated under odontogenic conditions (Table 1) and was predicted to be involved in TGFβ pathway (Fig. 4d). Automated western blot showed that miR-27a-5p mimics promoted odontogenic differentiation of DPSCs by upregulating the protein expressions of DSP, DMP-1, ALP, and RUNX2 (Fig. 7a), and activated TGFβ1/smads signaling pathway by increasing TGFβ1, TGFR1, p-Smad2/3, and Smad4 proteins (Fig. 7b).

**Fig. 8:**
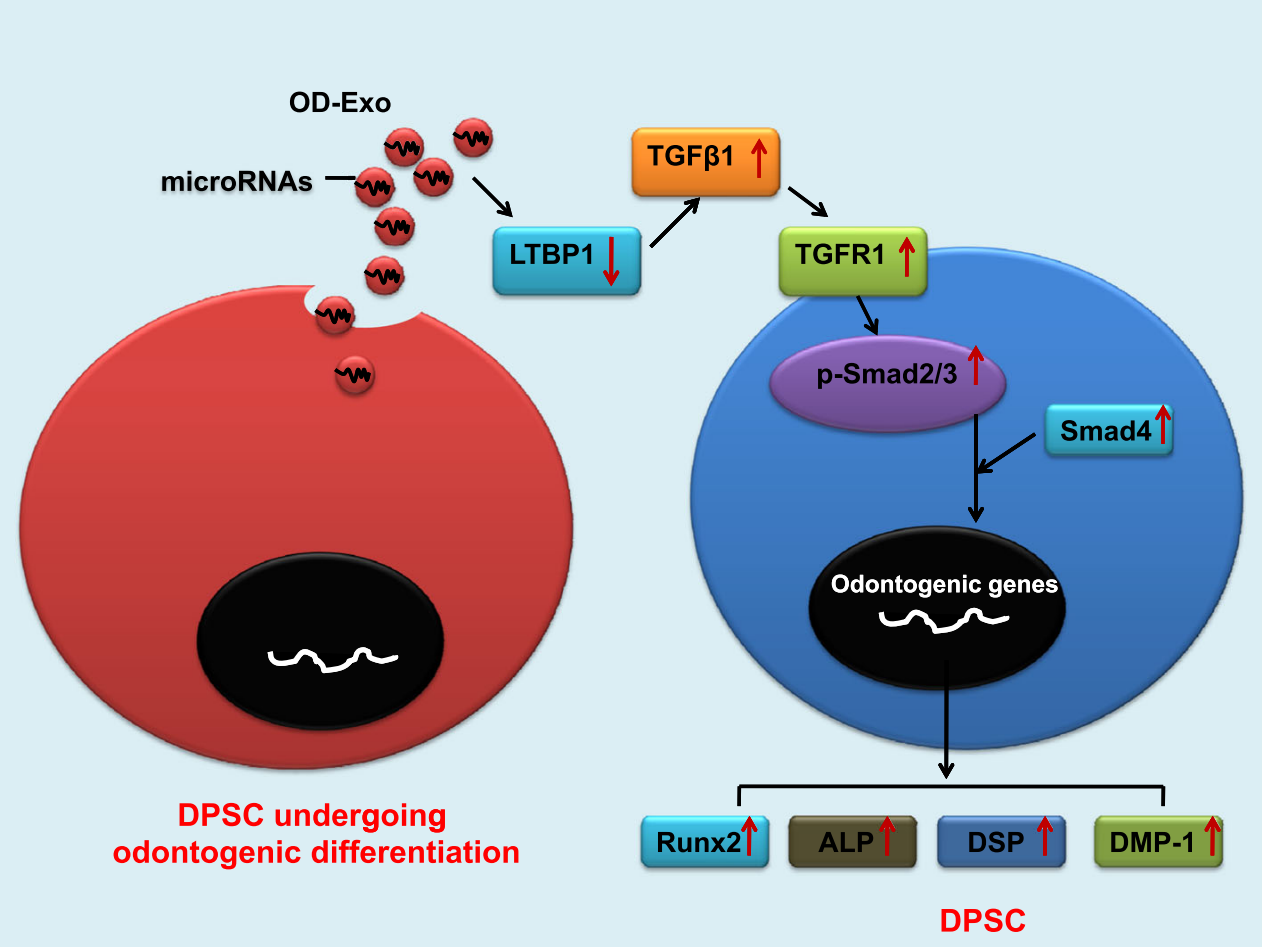
Summary of the function of OD-Exo in the odontogenic differentiation of DPSCs through TGFβ1/smads signaling pathway via the transfer of mircoRNAs

As is known, exosomal microRNAs can negatively regulate gene expression by binding to their target mRNAs through base pairing to the 3′-UTR, there may exist some other inhibitory molecules between the miR-27a-5p and TGFβ1 signaling. Latent TGF-β-binding protein 1 (LTBP1), one of the inhibitory molecules of TGFβ1 signaling, which form latent complexes with TGFβ by covalently binding the TGFβ propeptide (LAP) via disulfide bonds, plays a role in maintaining TGFβ latency by anchoring TGF-β to the extracellular matrix [19]. It was found that the mutation of LTBP-1 in mice resulted in excess active TGF-β, which caused increased signaling through its receptor and accumulation of nuclear p-Smad2/3 [20]. Using four publicly available bioinformatics tools, LTBP1 was found to be targeted by miR-27a-5p (Fig. 4b, d). We used western blot and double luciferase assay to test whether LTBP1 could be directly downregulated by miR-27a-5p. Western blot showed that LTBP1 was downregulated by miR-27a-5p mimics (Fig. 7c). Figure 7d showed the predicted miRNA binding sites in the 3′-UTR of LTBP1. The luciferase reporter assay was used to determine whether miR-27a-5p could target the 3′-UTR of LTBP1 directly. The 3′-UTR fragment (LTBP1-WT) of LTBP1 containing a miR-27a-5p binding site and mutant fragments (LTBP1-MUT) was cloned into luciferase reporter vectors. miR-27a-5p was found to significantly reduce LTBP1-WT luciferase activity while it had no effect on the luciferase activity of LTBP1-MUT group (Fig. 7e). Hence, the exosomal microRNAs promoted odontogenic differentiation via TGFβ1/smads signaling pathway by downregulating the inhibitory molecule LTBP1 (Fig. 8).

## Discussion

Regenerative endodontic treatment which has been defined as biologically based procedures designed to replace damaged structures, including dentin and root structures, as well as cells of the pulp–dentin complex, has great potential in treating endodontic disease [21]. Stem cells can be implanted into the root canal via a suitable medium, induced to proliferate, migrate, and differentiate into different cell types by biomaterials and growth factors to regenerate damaged tissue [22]. DPSCs is a suitable source for cells in regenerative endodontic treatment, and its differentiation into odontogenic lineage induced by biomaterials and growth factors is essential in dental pulp regeneration [1]. As reported, exosomes can be used as biomimetic tools to induce odontoblast-specific differentiation of DPSCs in regenerative endodontic treatment [14]. The present study determined that endocytosis of exosomes triggered odontogenic differentiation of DPSCs by evaluating the expression of ALP, RUNX2, odontoblast-specific marker DMP-1, and DSP proteins. Moreover, this study showed that exosomes isolated from DPSCs undergoing odontogenic differentiation (OD-Exo) were better inducers of DPSC differentiation than exosomes undergrowth medium (UN-Exo). Consistent with previous studies, cell type-specific exosomes can induce lineage-specific differentiation of stem cells. For example, DPSC-derived exosomes can direct the differentiation of the bone marrow MSCs towards an odontogenic lineage [14]. Similarly, osteoblast-derived exosomes containing instructive factors promoted the osteogenic differentiation of MSCs, while the adipocyte-derived exosomes triggered adipogenic differentiation of MSCs [7].

Exosomes, by carrying cell-type specific biological molecules such as protein, mRNA, and microRNA, serve as a mode of intercellular communications during tissue formation and repair [7]. Exosomal microRNAs can negatively regulate gene expression by binding to their target mRNAs through base pairing to the 3′-UTR, causing translational repression of the mRNA in recipient cells [23]. Exosomal microRNAs play important roles in stem cell differentiation [24]; however, the function of microRNAs encapsulated in exosomes derived from DPSCs is still unrevealed. In the present study, we performed microRNA sequencing to clarify microRNA expression profiles of exosomes derived from DPSCs. The results showed microRNA levels in OD-Exo were significantly changed when compared with that in UN-Exo. There were 28 microRNAs significantly changed in OD-Exo isolated under odontogenic conditions, of which 7 microRNAs increased (miR-5100, miR-27a-5p, miR-652-3p, miR-1260a, miR-1260b, let-7f-1-3p, and miR-370-3p) and 21 microRNAs decreased (miR-193a-5p, miR-4792, miR-505-3p, miR-629-5p, miR-140-3p, miR-185-5p, miR-146b-5p, miR-339-5p, miR-1246, miR-107, miR-320d, miR-451a, miR-215-5p, miR-126-3p, miR-3687, miR-31-5p, miR-210-3p, miR-1-3p, miR-10a-5p, miR-10b-5p, and miR-619-5p). Among these, 2 upregulated microRNAs (miR-5100 [25], miR-652-3p [26]), and 5 downregulated microRNAs (miR-185-5p [27], miR-107 [28], miR-215-5p [29], miR-31-5p [30], and miR-10b-5p [31]) have been found to play important roles in stem cell differentiation. miR-5100 has been verified to be upregulated during osteoblast differentiation and promoted osteogenic differentiation of MSCs [25]. miR-652-3p has been found significantly upregulated during neuronal differentiation and might play a pivotal role in the promotion of neuronal differentiation [26]. On the other hand, suppression of miR-185-5p enhanced ameloblast differentiation of LS8 cells and osteogenesis of MC3T3-E1 cells by regulating Dlx2 expression [27]. miR-107 was found to inhibit myoblast differentiation [28] and adipocyte differentiation [32]. miR-215-5p inhibited adipocyte differentiation of 3 T3-L1 cells through post-transcriptional regulation of fibronectin type III domain containing 3B (FNDC3B) and catenin-beta interacting protein 1 (CTNNBIP1) during early adipogenesis [29]. miR-31-5p inhibition enhanced the adipogenic differentiation in hADSCs, since miR-31-5p directly bound to the 3′-UTR of C/EBP-α to inhibit its expression [30]. Downregulation of miR-10b-5p promoted the differentiation of 3 T3-L1 cells and adipogenesis by upregulating the Apol6 expression [31]. In the present study, we transfected DPSCs with miR-27a-5p which was expressed 11 times higher in OD-Exo, and the results show miR-27a-5p mimics promoted odontogenic differentiation of DPSCs by upregulating the protein expressions of DSP, DMP-1, ALP, and RUNX2. Consequently, we can draw the conclusion that exosomes isolated from DPSCs undergoing odontogenic differentiation (OD-Exo) promoted the odontogenic differentiation via the transfer of mircoRNAs.

In our study, genes targeted by differentially expressed microRNAs were predicted by four publicly available bioinformatics tools (TargetScan, miRTarBase, miRDB, and miRWalk). Pathway analysis showed genes targeted by differentially expressed microRNAs were involved in multiple signal transductions, including TGFβ signaling pathway. TGFβ signaling has been shown to play important roles in odontogenic differentiation and tooth development [33, 34]. TGFβ signaling pathway was activated by exosomes via upregulating p-Smad2 and promoting nuclear localization of Smad4 [35]. Exosomes regulated the TGFβ pathway by increasing expression of Smad-3 in liver cells [36]. It has been revealed that exosomes regulated the TGFβ pathway by transferring exosomal microRNAs. Exosomal miR-302b influenced the TGFβ pathway by suppressing the expression of TGFβR2 [37]. Exosomal miR-let7c attenuated kidney injury by significantly downregulated TGFβ1 and TGFR1 in the kidneys [38]. Exosomal miR-132 promoted lymphangiogenic response by directly targeting Smad-7 and regulating TGF-β/Smad signaling [39]. In the present study, there were 16 genes targeted by 15 differentially expressed microRNAs were involved in TGFβ signaling. To confirm whether TGFβ signaling was activated by exosomal microRNAs, we evaluated the key proteins in TGFβ signaling. Consistent with pathway analysis, the protein expressions of TGFβ1, TGFR1, p-Smad2/3, and Smad4 were significantly increased when DPSCs were treated with OD-Exo for 48 h. In accordance, we inhibited the TGFβ1 signaling pathways by SB525334 and found protein levels of p-Smad2/3, DSP, and DMP-1 were significantly reduced in DPSCs during odontogenic differentiation induced by OD-Exo. We transfected DPSCs with miR-27a-5p mimics which was predicted to be involved in TGFβ pathway, and the results show miR-27a-5p mimics activated TGFβ1/smads signaling pathway by increasing TGFβ1, TGFR1, p-Smad2/3, and Smad4 proteins. Thus, the exosomal microRNAs promoted odontogenic differentiation via TGFβ1/smads signaling pathway.

Since exosomal microRNAs negatively regulate gene expression by binding to the 3′-UTRs of their target mRNAs, there may exist some inhibitory molecules between the miR-27a-5p and TGFβ1 signaling. As reported, latent TGF-β-binding protein 1 (LTBP1) was one of the inhibitory molecules of TGFβ1 signaling. The LTBP1 was first identified as forming latent complexes with TGFβ by covalently binding the TGFβ propeptide (LAP) via disulfide bonds and was thought to primarily play a role in maintaining TGFβ latency by anchoring TGF-β to the extracellular matrix [19]. Mutation of LTBP-1 in mice resulted in activating TGF-β signaling through its receptor and accumulation of nuclear p-Smad2/3 [20]. We performed western blot and double luciferase assay to test whether LTBP1 could be directly downregulated by miR-27a-5p. Interestingly, the western blot showed LTBP1 protein levels were reduced by miR-27a-5p mimics. Moreover, we found that miR-27a-5p could significantly reduce WT-LTBP1 luciferase activity and that miR-27a-5p had no effect on the luciferase activity of MUT-LTBP1 group. Luciferase reporter gene assay verified LTBP1 was the direct target gene of miR-27a-5p. Taken together, we draw a conclusion that the exosomal microRNAs promoted odontogenic differentiation via TGFβ1/smads signaling pathway by downregulating the inhibitory molecule LTBP1.

## Conclusions

This study identified the microRNA expression profiles of exosomes derived from DPSCs. Our results showed that OD-Exo isolated under odontogenic conditions were better inducers of DPSC differentiation. Exosomal microRNAs promoted odontogenic differentiation via TGFβ1/smads signaling pathway by downregulating the inhibitory molecule LTBP1.

## Additional files

Additional file 1: **Table S1.** Primer pairs used in the qRT-PCR. (DOCX 15 kb)

Additional file 2: **Table S2.** All genes targeted by differentially expressed microRNAs were analyze by four publicly available bioinformatics tools (TargetScan, miRTarBase, miRDB, and miRWalk). (XLSX 41 kb)

## Data Availability

The authors confirm that all data generated or analyzed during this study are available.

## Abbreviations

ALP: Alkaline phosphatase
DMP-1: Dentin matrix protein 1
DPSCs: Dental pulp stem cells
DSP: Dentin sialophosphoprotein
GO: Gene Ontology
LTBP1: Latent TGF-β-binding protein 1
MSCs: Mesenchymal stem cells
TGFR1: Transforming growth factor beta receptor 1
TGFβ1: Transforming growth factor beta 1
