# Supplementary material for: Lineage-specific exosomes promote the odontogenic differentiation of human dental pulp stem cells (DPSCs) through TGFβ1/smads signaling pathway via transfer of microRNAs
Source: Stem Cell Res Ther. 2019 Jun 13;10:170. doi: 10.1186/s13287-019-1278-x (PMC6567518; doi:10.1186/s13287-019-1278-x)
Supplement: Supplementary file 1 — Table S1. Primer pairs used in the qRT-PCR. (DOCX 15 kb) [file 13287_2019_1278_MOESM1_ESM.docx]

Table S1. Primer pairs used in the qRT-PCR.

| ID | Sequence (5’- 3’) |
| --- | --- |
| U6 F | CTCGCTTCGGCAGCACA |
| U6 R | AACGCTTCACGAATTTGCGT |
| All R | CTCAACTGGTGTCGTGGA |
| mir-5100 | TTCAGATCCCAGCGGTGCCTCT |
| mir-5100 RT | CTCAACTGGTGTCGTGGAGTCGGCAATTCAGTTGAGAGAGGCAC |
| mir-5100 F | ACACTCCAGCTGGGTTCAGATCCCAGCGGTGC |
| mir-1260a | ATCCCACCTCTGCCACCA |
| mir-1260a RT | CTCAACTGGTGTCGTGGAGTCGGCAATTCAGTTGAGTGGTGGCA |
| mir-1260a F | ACACTCCAGCTGGGATCCCACCTCTGCC |
| hsa-mir-210-3p RT | CTCAACTGGTGTCGTGGAGTCGGCAATTCAGTTGAGTCAGCCGC |
| hsa-mir-210-3p F | ACACTCCAGCTGGGCTGTGCGTGTGACAGCGG |
| hsa-mir-10b-5p RT | CTCAACTGGTGTCGTGGAGTCGGCAATTCAGTTGAGCACAAATT |
| hsa-mir-10b-5p F | ACACTCCAGCTGGGTACCCTGTAGAACCGAATT |
